# Whole-Genome Shotgun Sequencing of Cephalosporin-Resistant *Salmonella enterica* Serovar Typhi

**DOI:** 10.1128/genomeA.01639-16

**Published:** 2017-03-09

**Authors:** Camilla Rodrigues, Arti Kapil, Anita Sharma, Naveen Kumar Devanga Ragupathi, Francis Yesurajan Inbanathan, Balaji Veeraraghavan, Gagandeep Kang

**Affiliations:** a Hinduja Hospital, Mumbai, India; b Department of Microbiology, AIIMS, New Delhi, India; c Department of Lab Medicine, Fortis Hospital, Mohali, India; d Department of Clinical Microbiology, Christian Medical College, Vellore, India; e Wellcome Trust Research Laboratory and Division of Gastrointestinal Sciences, Christian Medical College, Vellore, India

## Abstract

Typhoid is one of the leading causes of mortality in developing countries. Here, we report the draft genome sequences of four *Salmonella enterica* serovar Typhi strains isolated from bloodstream infections in a tertiary care hospital. The sequence data indicate genomes of ~4.5 Mb for all isolates, with one plasmid in each.

## GENOME ANNOUNCEMENT

Resistance to third-generation cephalosporins is increasing in *Salmonella* spp. due to CTX-M-type extended-spectrum cephalosporin (ESC) resistance, as well as production of acquired AmpC β-lactamases and extended-spectrum β-lactamases (ESBLs) (1, 2).

In this study, we present the draft genome sequences of four *Salmonella enterica* serovar Typhi strains (429038, 430040, 7830, and 458426) isolated from blood. To further understand the mechanism behind cephalosporin resistance, the whole-genome sequences (WGSs) of these isolates were analyzed. The Ion Torrent PGM platform was used to perform WGS with 400-bp chemistry. Raw data assembly was achieved *de novo* in the SPAdes assembler version 5.0.0.0 embedded in the Torrent suite server version 5.0.3. Upon assembly, the genomes of isolates 429038, 430040, 7830, and 458426 showed 65, 61, 58, and 58 contigs (≥500 bp), respectively, and the genome coverage of these isolates ranged from 51× to 62×. Sequence annotation was done using PATRIC, the bacterial bioinformatics database and analysis resource (http://www.patricbrc.org) (3), RAST, the Rapid Annotations using Subsystems Technology server (http://rast.nmpdr.org) (4, 5), and PGAP, the NCBI Prokaryotic Genome Annotation Pipeline (http://www.ncbi.nlm.nih.gov/genomes/static/Pipeline.html).

The complete details about the four genomes are given in Table 1. From the genomes of 429038, 430040, 7830, and 458426, the annotations revealed 22, 22, 20, and 22 antimicrobial resistance genes from the ARDB database, and 58, 60, 56, and 59 from the CARD database, respectively. Analysis with CRISPRFinder resulted in two confirmed clustered regularly interspaced short palindromic repeats for isolate 7830 and one each in the genomes of isolates 429038, 430040, and 458426 (6).

**TABLE 1:** Whole-genome characterization of *Salmonella* Typhi isolates from clinical specimens^a^

Isolate ID	Sequence type	Yr of isolation	Total size (bp)	CDSs, rRNAs, tRNAs	Coverage (×)	Contigs (≥500 bp)	CRISPRs	Antimicrobial resistance gene(s)	Plasmid	Accession no.
429038	1	2016	4,801,759	5,234, 10, 58	61	65	1	*bla*_SHV-12_, *qnr*B7	IncX3	MQUL00000000
430040	1	2016	4,798,259	4,263, 11, 60	51	61	1	*bla*_SHV-12_, *qnr*B7	IncX3	MQUM00000000
7830	592	2016	4,831,725	5,151, 14, 67	58	58	2	*bla*_CMY-2_	IncA/C2	MQUN00000000
458426	1	2016	4,796,415	5,237, 11, 59	61	58	1	*bla*_SHV-12_, *qnr*B7	IncX3	MQUO00000000

^a^ CDSs, coding sequences; CRISPRs, clustered regularly interspaced short palindromic repeats.

Further downstream analysis with the MLST version 1.8 tool (https://cge.cbs.dtu.dk/services/MLST) (7) revealed information on the isolates’ sequence type (ST). Isolates 429038, 430040, and 458426 belong to ST-1, while 7830 belongs to ST-592. Analysis with ResFinder version 2.1 revealed antimicrobial resistance genes *bla*_SHV-12_ and *qnr*B7 for 429038, 430040, and 458426, while 7830 had the *bla*_CMY-2 _gene. This confirmed the mechanism of cephalosporin resistance in these isolates. In addition, isolates 429038, 430040, and 458426 were found to harbor the IncX3 plasmid, but isolate 7830 had IncA/C2, as determined with PlasmidFinder version 1.3 (http://www.cbs.dtu.dk/services).

The identification of genes for cephalosporin resistance in *Salmonella* Typhi should lead to continuous surveillance of plasmids and the spread of antimicrobial resistance in developing countries like India.

### Accession number(s).

The whole-genome sequences of the four *Salmonella* Typhi isolates 429038, 430040, 7830, and 458426 were deposited in DDBJ/ENA/GenBank under the accession numbers mentioned in Table 1. The versions described in this paper are the first versions.
